# Synthesis and crystal structure of bis­[(1*E*,6*E*)-1,7-bis­(4-acet­yloxy-3-meth­oxy­phen­yl)hepta-1,6-diene-3,5-dionato(1−)-κ^2^*O*,*O*′](methanol)dioxidouranium(VI) toluene monosolvate

**DOI:** 10.1107/S2056989026002525

**Published:** 2026-04-02

**Authors:** Van Ha Nguyen, Thi Nguyet Trieu, Chien Thang Pham

**Affiliations:** a Faculty of Chemistry, VNU University of Science, Vietnam National University, Hanoi, 19 Le Thanh Tong, Hanoi, Vietnam; Illinois State University, USA

**Keywords:** curcumin, 4,4′-di­acetyl­curcumin, uranyl complex, crystal structure

## Abstract

The first uranium–curcuminoid coordination compound has been synthesized and structurally characterized. The title complex, [U(O)_2_(C_25_H_23_O_8_)_2_(CH_3_OH)]·C_6_H_5_CH_3_, crystallizes in the monoclinic space group *C*2/*c*. The neutral complex comprises a uran­yl(VI) unit ({O=U=O}^2+^) coordinated by two monoanionic bidentate 4,4′-di­acetyl­curcuminato ligands (C_25_H_23_O_8_) and one methanol co-ligand, resulting in a distorted penta­gonal–bipyramidal coordination geometry. The O atoms from the *β*-diketonate moieties and the methanol mol­ecule form the equatorial plane, while the uranyl O atoms occupy the axial positions. In the crystal, O—H⋯O hydrogen bonds generate *R*_2_^2^(26) ring motifs, forming zigzag chains along the *a*-axis direction. Additional weak C—H⋯O inter­actions further consolidate the crystal packing through inter­chain aggregation.

## Abstract 2

The first uranium–curcuminoid complex, [U(C_25_H_23_O_8_)_2_O_2_(CH_3_OH)]·C_6_H_5_CH_3_, features a uran­yl(VI) center coordinated by two monoanionic bidentate 4,4′-di­acetyl­curcuminato ligands and one methanol mol­ecule. The uranium atom adopts a distorted penta­gonal–bipyramidal coordination geometry, with four O atoms from the *β*-diketonate moieties and one O atom from the methanol ligand defining the equatorial plane, while the two uranyl O atoms occupy the axial positions.

## 1. Chemical context

Curcumin [1,7-bis­(4-hy­droxy-3-meth­oxy­phen­yl)-1,6-hepta­diene-3,5-dione] is a major constituent of turmeric (*Curcuma longa*, Zingiberaceae) (Goel *et al.*, 2008 ▸). Beyond its widespread use as a spice and natural food coloring, turmeric has been employed in traditional medicine to treat a broad spectrum of diseases (Goel *et al.*, 2008 ▸; Esatbeyoglu *et al.*, 2012 ▸). The therapeutic potential of curcumin has attracted considerable attention, and numerous studies have confirmed its anti­oxidant, anti-inflammatory (Menon *et al.*, 2007 ▸; Dehzad *et al.*, 2023 ▸), anti­carcinogenic (Salem *et al.*, 2014 ▸), and anti­microbial (Dai *et al.*, 2022 ▸) properties. From a chemical perspective, curcumin and its structural analogues are natural *β*-diketone ligands capable of chelating and forming stable complexes with a wide range of metal ions, including main group, transition, and rare-earth metals (Bhagat *et al.*, 2025 ▸). In recent decades, metal-curcumin complexes have gained significant inter­est because of their diverse biological activities (Banerjee *et al.*, 2015 ▸; Prasad *et al.*, 2021 ▸; Bhagat *et al.*, 2025 ▸). However, their application is often limited by extremely poor solubility in water and in most common organic solvents (Wanninger *et al.*, 2015 ▸; Prasad *et al.*, 2021 ▸). To overcome this limitation, structural modifications such as etherification or esterification of curcumin have been developed, leading to various derivatives and a number of structurally characterized metal complexes (Wang *et al.*, 2014 ▸; Meza-Morales *et al.*, 2019 ▸; Pham *et al.*, 2020 ▸; Meza-Morales *et al.*, 2023*a* ▸). Nevertheless, comprehensive structural data on curcuminoid complexes remain limited, and no actinide-curcumin complex has hitherto been structurally characterized. Herein, we report the synthesis and crystal structure of the first uranyl complex with acetyl­ated curcumin (4,4′-di­acetyl­curcumin, **HL**).
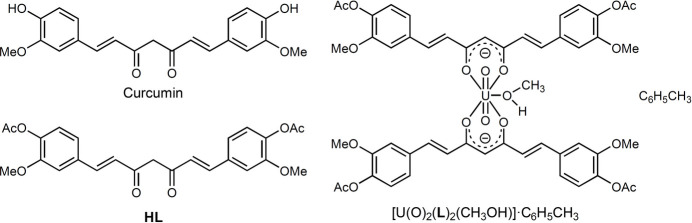


## 2. Structural commentary

**Figure 1:**
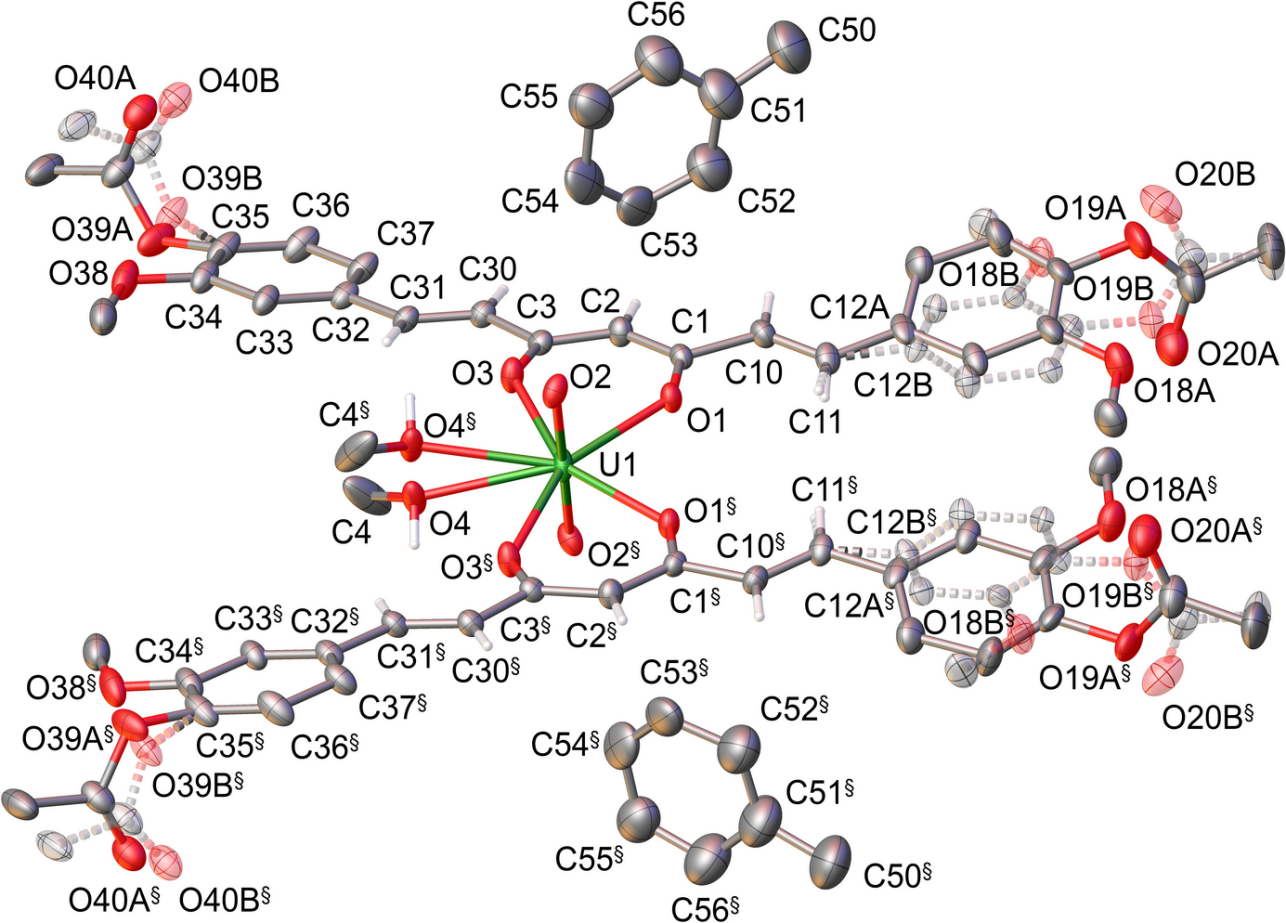
The mol­ecular structure of the title compound, with displacement ellipsoids drawn at the 50% probability level. Hydrogen atoms bonded to aromatic rings and methyl groups are omitted for clarity. Symmetry code: (§) −*x* + 1, *y*, −*z* + 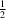.

The title compound crystallizes in the centrosymmetric monoclinic space group *C*2/*c*, with half of the mol­ecule, [U(O)_2_(**L**)_2_(CH_3_OH)]·C_6_H_5_CH_3_, in the asymmetric unit (Fig. 1 ▸). The complex consists of one uranyl unit (UO_2_^2+^), two monodeprotonated acetyl­ated curcumin ligands {**L**}^−^, and one methanol co-ligand. The methanol mol­ecule is disordered over two symmetry-related sites with equal occupancy factors of 0.5. The uranium atom adopts a distorted penta­gonal–bipyramidal coordination geometry, with the oxido ligands occupying the axial positions. The {**L**}^−^ ligands equatorially coordinate through (*O*,*O*)-chelating *β*-diketonate moieties, while the equatorial plane is completed by a disordered methanol ligand. The uranium atom lies 0.064 (6) Å out of the mean equatorial plane. The U=O bond length [1.772 (6) Å] and O=U=O bond angle [179.5 (3)°] fall within the expected range (Ainscough *et al.*, 1998 ▸, Huuskonen *et al.*, 2007 ▸, Al-Anber *et al.*, 2011 ▸). The equatorial U1—O bond distances [U1—O1 = 2.345 (5) Å and U1—O3 = 2.351 (5) Å] are comparable to those reported for penta­gonal-bipyramidal *β*-diketonate uranyl complexes (Hernandez *et al.*, 2022 ▸; Monzón González *et al.*, 2024 ▸; Jabborova *et al.*, 2024 ▸). The U—O_MeOH_ bond distance [U1—O4 = 2.567 (9) Å] is longer than the U—O**_L_** bonds, indicating weaker coordination of the solvent mol­ecule compared to the chelating *β*-diketonate ligands. The C—C and C—O bond lengths within the chelate rings are consistent with those observed in related complexes of **HL** with other divalent metal ions (Meza-Morales *et al.*, 2019 ▸; Pham *et al.*, 2020 ▸). The partial double-bond character of these bonds reflects the expected π-electron delocalization within the *β*-diketonate moieties. Peripheral portions of the {**L**}^−^ ligand are disordered over two positions, with refined occupancy factors of 0.5083 (1)/0.4916 (9) for one aromatic ring and its acetyl group, and 0.6046 (3)/0.3953 (7) for the acetyl group on the other ligand fragment.

## 3. Supra­molecular features

In the crystal structure, the complex does not form columnar packing or significant π–π stacking inter­actions. The mol­ecules are arranged as discrete units, resulting in solvent-accessible voids. Void analysis performed using *OLEX2* (Dolomanov *et al.*, 2009 ▸) indicates that the structure occupies 3588.18 Å^3^ (63.31%) of the unit-cell volume, leaving void space that is occupied by toluene solvent mol­ecules. These solvent mol­ecules contribute to the overall cohesion of the crystal structure.

**Figure 2:**
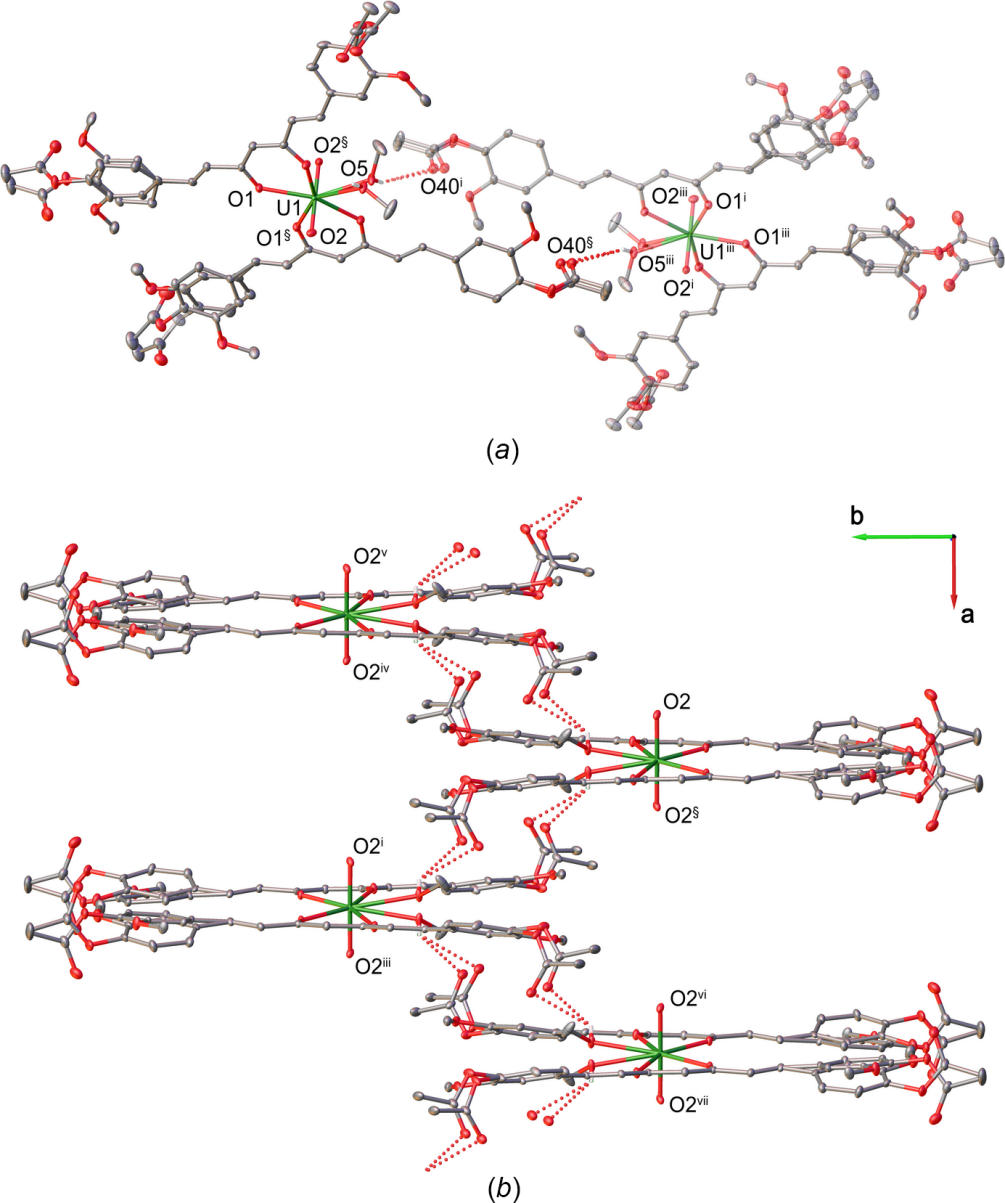
(*a*) Mol­ecular packing diagram showing the 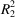
(26) hydrogen-bonding motif. (*b*) Polymeric chains extending along the *a*-axis direction. Hydrogen bonds are shown as dashed lines. Solvent mol­ecules and hydrogen atoms not involved in hydrogen bonding have been omitted for clarity. Symmetry codes: (§) −*x* + 1, *y*, −*z* + 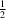; (i) *x* + 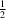, −*y* + 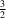, *z* − 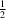; (iii) −*x* + 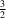, −*y* + 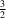, −*z*; (iv) −*x* + 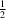, −*y* + 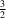, −*z* + 1; (v) *x* − 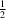, −*y* + 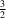, *z* + 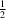; (vi) *x* + 1, *y*, *z* − 1; (vii) −*x* + 2, *y*, −*z* − 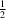.

**Figure 3:**
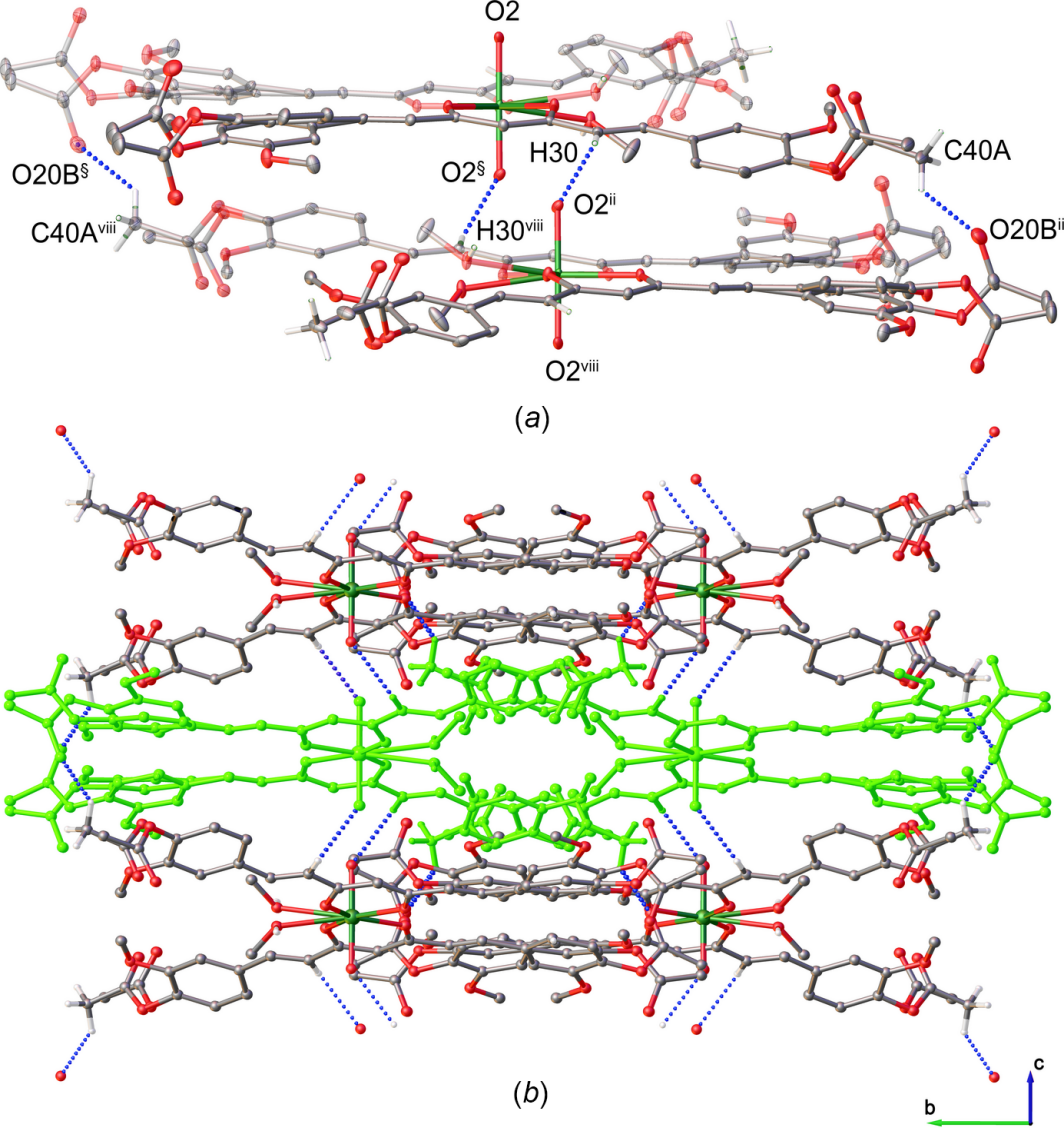
(*a*) Mol­ecular packing diagram showing weak C—H⋯O hydrogen bonds between units in adjacent chains. Symmetry codes: (§) −*x* + 1, *y*, −*z* + 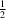; (iii) *x*, −*y* + 1, *z* + 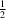; (viii) −*x* + 1, −*y* + 1, −*z* + 1. (*b*) Crystal packing viewed along the *a*-axis direction illustrating the aggregation of chains. The central chain is highlighted for clarity. Hydrogen bonds are shown as dashed lines. Solvent mol­ecules and hydrogen atoms not involved in weak hydrogen bonds have been omitted for clarity.

In the crystal, O—H⋯O hydrogen bonds between the hydroxyl groups of the disordered methanol mol­ecules and the carbonyl O40 atoms of adjacent units link the mol­ecules into inversion dimers via 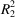
(26) hydrogen-bonding motifs (Fig. 2 ▸*a*, Table 1 ▸). These hydrogen bonds further connect the dimers into zigzag chains extending along the *a*-axis direction (Fig. 2 ▸*b*). In addition, weak C40*A*—H40*D*⋯O20*B* hydrogen bonds (Fig. 3 ▸*a*, Table 1 ▸) link the chains into a three-dimensional supra­molecular network (Fig. 3 ▸*b*). A further weak inter­molecular C30—H30⋯O2 contact also contributes to the consolidation of the crystal packing.

## 4. Database survey

A search of the Cambridge Structural Database (CSD version 6.00, update on August 2025; Groom *et al.*, 2016 ▸) resulted in 25 entries describing homoleptic metal complexes of curcumin and its derivatives. Among these, ten structures correspond to coordination compounds derived from 4,4′-di­acetyl­curcumin, including HOBDUA, JOCQEA, JOCQUQ, JOCRAX, PEJREE (Meza-Morales *et al.*, 2019 ▸), KUNTUL, KUNVAT, KUNVEX, KUNVIB (Pham *et al.*, 2020 ▸) and YIHKIN (Meza-Morales *et al.*, 2023*b* ▸). A separate search for uranyl complexes based on *β*-diketone ligands returned 93 entries exhibiting penta­gonal–bipyramidal geometries similar to that observed in the title structure. Of these, fourteen structures have been reported within the past decade, including BUHDEP (Ma *et al.*, 2015 ▸), NOVBUX (Kawasaki *et al.*, 2015 ▸), VOWCUH (Vats *et al.*, 2015 ▸), CIVVAH and CIVVEL (Carter *et al.*, 2018 ▸), XEXZOS and XEXZUY (Kurzajewska *et al.*, 2018 ▸), TAMTUA (Hernandez *et al.*, 2022 ▸), EFOGOZ (Monzón González *et al.*, 2024 ▸), GUGREJ (Jabborova *et al.*, 2024 ▸), IMICEQ and IMICIU (Tafeenko *et al.*, 2025 ▸), LAFKAJ and VACCAI (Clark *et al.*, 2025 ▸).

## 5. Synthesis and crystallization

4,4′-Di­acetyl­curcumin (90.4 mg, 0.2 mmol) was added to 1.55 mL solution of UO_2_(OAc)_2_·2H_2_O (42.4 mg, 0.1 mmol) in MeOH. The color of the reaction mixture immediately changed from yellow to red–orange. After stirring the reaction mixture for 15 min, two drops of Et_3_N were added. Then, the temperature was increased to 313 K and kept for 1 h. During this process, a red–orange precipitate deposited, which was filtered off, washed with a small amount of MeOH and dried under vacuum. Single crystals suitable for X-ray analysis were obtained by slow evaporation of a solution of the complex in a mixture of CH_2_Cl_2_, MeOH and toluene. Yield: ∼70% (82 mg).

IR (KBr, cm^−1^): 3448 (*br*, *m*), 3005 (*w*), 2942 (*w*), 1764 (*m*), 1722 (*m*), 1627 (*m*), 1599 (*m*), 1511 (*s*), 1467 (*m*), 1394 (*m*), 1295 (*m*), 1259 (*m*), 1198 (*m*), 1156 (*m*), 1121 (*m*), 1031 (*w*), 985 (*w*), 905 (*m*), 849 (*w*), 606 (*w*), 466 (*w*).

^1^H NMR (500 MHz, CDCl_3_, ppm): 8.11 (*br*, *d*, *J* = 15.0 Hz, 2 H, CH), 7.62 (*d*, *J* = 16.0 Hz, 2 H, CH), 7.17–7.05 (*m*, 12 H, Ph), 6.98 (*d*, *J* = 15.5 Hz, 2 H, CH), 6.57 (*d*, *J* = 15.5 Hz, 2 H, CH), 6.01 (*s*, 1 H, C_α_H), 5.87 (*s*, 1 H, C_α_H), 3.88 (*s*, 6 H, OCH_3_), 3.78 (*s*, 6 H, OCH_3_), 2.36 (*s*, 6 H, CH_3_COO), 2.33 (*s*, 6 H, CH_3_).

## 6. Refinement

Crystal data, data collection and structure refinement details are summarized in Table 2 ▸. The aromatic ring (C12–C17) and its acetyl group are disordered over two positions with refined occupancies of 0.5083 (1):0.4916 (9); another acetyl group is disordered in a 0.6046 (3):0.3953 (7) ratio. Aromatic C atoms of the toluene solvent were restrained to be approximately isotropic (ISOR) and planar (FLAT). Bond distances C11—C12*A*, C15*A*—O19*A* and those within the toluene ring were restrained using DFIX 1.4, while equivalent C—C distances in disordered acetyl groups and toluene ring were constrained using SADI. Displacement ellipsoids of disordered atom pairs including (C20*A*, C20*B*), (C39*A*, C39*B*), (C40*A*, C40*B*) and (O40*A*, O40*B*) were restrained to be similar (EADP). The *U*_ij_ values of disordered atoms and aromatic carbon atoms of the toluene solvent we restrained using RIGU. Hydrogen atoms were placed in calculated positions and refined using a riding model with isotropic displacement parameters based on those of the parent atom [C—H = 0.95 Å, *U*_iso_(H) = 1.2*U*_eq_C for CH; C—H = 0.98 Å, *U*_iso_(H) = 1.5*U*_eq_C for CH_3_; O—H = 0.84 Å, *U*_iso_(H) = 1.5*U*_eq_O for OH]. Two reflections, (131) and (243), were omitted owing to poor agreement between observed and calculated intensities.

## Supplementary Material

Crystal structure: contains datablock(s) I. DOI: 10.1107/S2056989026002525/ej2019sup1.cif

CCDC reference: 2536300

Additional supporting information:  crystallographic information; 3D view; checkCIF report

## Figures and Tables

**Table 1:** Hydrogen-bond geometry (Å, °)

*D*—H⋯*A*	*D*—H	H⋯*A*	*D*⋯*A*	*D*—H⋯*A*
O4—H4⋯O40*A*^i^	0.84	2.33	2.99 (2)	136
O4—H4⋯O40*B*^i^	0.84	2.67	3.23 (4)	125
C40*A*—H40*D*⋯O20*B*^ii^	0.98	2.46	3.25 (4)	137
C30—H30⋯O2^ii^	0.95	2.58	3.436 (9)	150

Symmetry codes: (i) 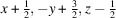; (ii) 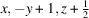.

**Table 2:** Experimental details

Crystal data	
Chemical formula	[U(C_25_H_23_O_8_)_2_O_2_(CH_4_O)]·C_7_H_8_
*M*_r_	1297.07
Crystal system, space group	Monoclinic, *C*2/*c*
Temperature (K)	170
*a*, *b*, *c* (Å)	15.392 (4), 23.149 (6), 15.907 (4)
β (°)	90.577 (9)
*V* (Å^3^)	5668 (3)
*Z*	4
Radiation type	Mo *K*α
μ (mm^−1^)	2.94
Crystal size (mm)	0.25 × 0.18 × 0.12

Data collection	
Diffractometer	Bruker APEXII CCD
Absorption correction	Multi-scan (*SADABS*; Krause *et al.*, 2015 ▸)
*T*_min_, *T*_max_	0.595, 0.745
No. of measured, independent and observed [*I* > 2σ(*I*)] reflections	35282, 5389, 4061
*R*_int_	0.080
(sin θ/λ)_max_ (Å^−1^)	0.613

Refinement	
*R*[*F*^2^ > 2σ(*F*^2^)], *wR*(*F*^2^), *S*	0.054, 0.147, 1.12
No. of reflections	5389
No. of parameters	519
No. of restraints	561
H-atom treatment	H-atom parameters constrained
Δρ_max_, Δρ_min_ (e Å^−3^)	1.50, −0.85

Computer programs: *APEX2* and *SAINT* (Bruker, 2014 ▸), *SHELXT* (Sheldrick, 2015*a* ▸), *SHELXL2018/3* (Sheldrick, 2015*b* ▸) and *OLEX2* (Dolomanov *et al.*, 2009 ▸).

## supplementary crystallographic information

### Bis[(1E,6E)-1,7-bis(4-acetyloxy-3-methoxyphenyl)hepta-1,6-diene-3,5-dionato(1-)-κ2O,O'](methanol)dioxidouranium(VI) toluene monosolvate Crystal data

**Table:**

[U(C_25_H_23_O_8_)_2_O_2_(CH_4_O)]·C_7_H_8_	*F*(000) = 2600
*M**_r_* = 1297.07	*D*_x_ = 1.520 Mg m^−^^3^
Monoclinic, *C*2/*c*	Mo *K*α radiation, λ = 0.71073 Å
*a* = 15.392 (4) Å	Cell parameters from 9952 reflections
*b* = 23.149 (6) Å	θ = 3.0–25.8°
*c* = 15.907 (4) Å	µ = 2.94 mm^−^^1^
β = 90.577 (9)°	*T* = 170 K
*V* = 5668 (3) Å^3^	Block, dark orange
*Z* = 4	0.25 × 0.18 × 0.12 mm

### Bis[(1E,6E)-1,7-bis(4-acetyloxy-3-methoxyphenyl)hepta-1,6-diene-3,5-dionato(1-)-κ2O,O'](methanol)dioxidouranium(VI) toluene monosolvate Data collection

**Table:**

Bruker APEXII CCD diffractometer	4061 reflections with *I* > 2σ(*I*)
φ and ω scans	*R*_int_ = 0.080
Absorption correction: multi-scan (SADABS; Krause *et al.*, 2015)	θ_max_ = 25.8°, θ_min_ = 3.0°
*T*_min_ = 0.595, *T*_max_ = 0.745	*h* = −16→18
35282 measured reflections	*k* = −28→28
5389 independent reflections	*l* = −19→19

### Bis[(1E,6E)-1,7-bis(4-acetyloxy-3-methoxyphenyl)hepta-1,6-diene-3,5-dionato(1-)-κ2O,O'](methanol)dioxidouranium(VI) toluene monosolvate Refinement

**Table:**

Refinement on *F*^2^	Primary atom site location: dual
Least-squares matrix: full	Hydrogen site location: inferred from neighbouring sites
*R*[*F*^2^ > 2σ(*F*^2^)] = 0.054	H-atom parameters constrained
*wR*(*F*^2^) = 0.147	*w* = 1/[σ^2^(*F*_o_^2^) + (0.0669*P*)^2^ + 25.8409*P*] where *P* = (*F*_o_^2^ + 2*F*_c_^2^)/3
*S* = 1.12	(Δ/σ)_max_ < 0.001
5389 reflections	Δρ_max_ = 1.50 e Å^−^^3^
519 parameters	Δρ_min_ = −0.85 e Å^−^^3^
561 restraints	

### Bis[(1E,6E)-1,7-bis(4-acetyloxy-3-methoxyphenyl)hepta-1,6-diene-3,5-dionato(1-)-κ2O,O'](methanol)dioxidouranium(VI) toluene monosolvate Special details

**Table:**

Geometry. All esds (except the esd in the dihedral angle between two l.s. planes) are estimated using the full covariance matrix. The cell esds are taken into account individually in the estimation of esds in distances, angles and torsion angles; correlations between esds in cell parameters are only used when they are defined by crystal symmetry. An approximate (isotropic) treatment of cell esds is used for estimating esds involving l.s. planes.

### Bis[(1E,6E)-1,7-bis(4-acetyloxy-3-methoxyphenyl)hepta-1,6-diene-3,5-dionato(1-)-κ2O,O'](methanol)dioxidouranium(VI) toluene monosolvate Fractional atomic coordinates and isotropic or equivalent isotropic displacement parameters (Å^2^)

**Table:**

	*x*	*y*	*z*	*U*_iso_*/*U*_eq_	Occ. (<1)
U1	0.500000	0.50545 (2)	0.250000	0.05680 (18)	
O2	0.4046 (4)	0.5058 (2)	0.1867 (3)	0.0668 (14)	
O1	0.4467 (4)	0.42789 (19)	0.3290 (3)	0.0598 (13)	
O3	0.4215 (4)	0.5436 (2)	0.3633 (3)	0.0702 (15)	
O38	0.3369 (5)	0.8280 (3)	0.5497 (5)	0.101 (2)	
C1	0.4172 (5)	0.4220 (3)	0.4034 (5)	0.0526 (16)	
C3	0.4021 (5)	0.5249 (3)	0.4367 (4)	0.0518 (16)	
C10	0.4020 (5)	0.3630 (3)	0.4337 (5)	0.065 (2)	
H10	0.372203	0.359431	0.485431	0.078*	
C31	0.3808 (5)	0.6236 (3)	0.4885 (5)	0.0565 (18)	
H31	0.393299	0.635888	0.432905	0.068*	
C2	0.3978 (6)	0.4674 (3)	0.4561 (5)	0.064 (2)	
H2	0.379594	0.457766	0.511265	0.077*	
C32	0.3639 (5)	0.6697 (3)	0.5490 (5)	0.0613 (19)	
C30	0.3811 (5)	0.5683 (3)	0.5006 (5)	0.0573 (18)	
H30	0.366567	0.554765	0.555099	0.069*	
C33	0.3596 (6)	0.7257 (3)	0.5195 (6)	0.068 (2)	
H33	0.368408	0.733321	0.461522	0.081*	
C11	0.4247 (6)	0.3157 (3)	0.3977 (5)	0.067 (2)	
H11	0.456935	0.317462	0.347134	0.081*	0.508 (10)
H11A	0.448394	0.321125	0.343338	0.081*	0.492 (10)
C37	0.3525 (6)	0.6591 (4)	0.6338 (5)	0.076 (2)	
H37	0.356872	0.620859	0.654994	0.091*	
C35	0.3296 (6)	0.7600 (4)	0.6582 (6)	0.087 (3)	
C36	0.3347 (7)	0.7049 (5)	0.6883 (6)	0.092 (3)	
H36	0.326007	0.697645	0.746387	0.110*	
C34	0.3424 (6)	0.7714 (4)	0.5748 (6)	0.079 (2)	
C38	0.3590 (8)	0.8419 (4)	0.4648 (9)	0.105 (3)	
H38A	0.326945	0.816480	0.426151	0.158*	
H38B	0.421559	0.836469	0.456974	0.158*	
H38C	0.343645	0.882193	0.453055	0.158*	
C13B	0.3955 (14)	0.2404 (9)	0.4996 (15)	0.068 (5)	0.492 (10)
H13B	0.379701	0.270341	0.537332	0.081*	0.492 (10)
C14A	0.4087 (18)	0.1541 (10)	0.423 (2)	0.082 (5)	0.508 (10)
C15A	0.3589 (15)	0.1477 (8)	0.4939 (16)	0.080 (5)	0.508 (10)
C14B	0.3915 (13)	0.1834 (8)	0.5236 (13)	0.067 (5)	0.492 (10)
C17A	0.3467 (18)	0.2500 (10)	0.4994 (17)	0.095 (7)	0.508 (10)
H17A	0.319308	0.282185	0.524921	0.114*	0.508 (10)
C13A	0.432 (2)	0.2090 (11)	0.3961 (18)	0.076 (6)	0.508 (10)
H13A	0.471153	0.211618	0.350243	0.091*	0.508 (10)
C12A	0.403 (3)	0.2611 (9)	0.432 (3)	0.076 (7)	0.508 (10)
O19A	0.3334 (13)	0.0913 (6)	0.5193 (13)	0.113 (6)	0.508 (10)
C15B	0.4158 (17)	0.1413 (9)	0.4687 (17)	0.071 (5)	0.492 (10)
C16B	0.4473 (15)	0.1547 (8)	0.3931 (16)	0.069 (5)	0.492 (10)
H16B	0.468259	0.124917	0.357690	0.083*	0.492 (10)
O19B	0.4177 (12)	0.0840 (6)	0.4973 (12)	0.105 (5)	0.492 (10)
O18A	0.4415 (11)	0.1047 (6)	0.3896 (10)	0.105 (5)	0.508 (10)
C18A	0.4869 (17)	0.1098 (10)	0.3160 (15)	0.112 (8)	0.508 (10)
H18A	0.467598	0.144500	0.285845	0.167*	0.508 (10)
H18B	0.476328	0.075701	0.280881	0.167*	0.508 (10)
H18C	0.549152	0.112882	0.328795	0.167*	0.508 (10)
O18B	0.3646 (12)	0.1657 (6)	0.6016 (10)	0.106 (6)	0.492 (10)
C12B	0.421 (3)	0.2523 (11)	0.425 (3)	0.067 (9)	0.492 (10)
C18B	0.3392 (17)	0.2079 (9)	0.6574 (14)	0.104 (8)	0.492 (10)
H18D	0.297215	0.233722	0.629780	0.156*	0.492 (10)
H18E	0.390035	0.230257	0.675735	0.156*	0.492 (10)
H18F	0.312142	0.189845	0.706314	0.156*	0.492 (10)
C16A	0.327 (2)	0.1922 (9)	0.5336 (18)	0.110 (8)	0.508 (10)
H16A	0.292933	0.187613	0.582515	0.132*	0.508 (10)
O20B	0.2817 (14)	0.0707 (10)	0.4557 (15)	0.144 (8)	0.492 (10)
O20A	0.4621 (13)	0.0733 (7)	0.5756 (13)	0.127 (6)	0.508 (10)
C19B	0.350 (2)	0.0521 (13)	0.491 (2)	0.115 (8)	0.492 (10)
C19A	0.389 (2)	0.0561 (10)	0.554 (2)	0.116 (8)	0.508 (10)
O39B	0.303 (3)	0.7992 (17)	0.722 (3)	0.089 (8)	0.40 (2)
O40B	0.169 (2)	0.7907 (13)	0.688 (2)	0.095 (4)	0.40 (2)
C39B	0.228 (3)	0.8160 (16)	0.730 (3)	0.086 (7)	0.40 (2)
C40B	0.212 (3)	0.8665 (18)	0.784 (3)	0.126 (9)	0.40 (2)
H40A	0.218743	0.854929	0.843348	0.189*	0.40 (2)
H40B	0.153465	0.881114	0.774306	0.189*	0.40 (2)
H40C	0.254641	0.896842	0.771628	0.189*	0.40 (2)
O4	0.5292 (8)	0.6146 (4)	0.2494 (12)	0.080 (5)	0.5
H4	0.575033	0.615103	0.221182	0.120*	0.5
C4	0.540 (2)	0.6518 (13)	0.3234 (19)	0.176 (16)	0.5
H4A	0.545639	0.692077	0.305402	0.264*	0.5
H4B	0.592321	0.640222	0.354705	0.264*	0.5
H4C	0.489167	0.647879	0.359607	0.264*	0.5
C40A	0.2551 (17)	0.8910 (12)	0.761 (2)	0.126 (9)	0.60 (2)
H40D	0.283804	0.884775	0.815389	0.189*	0.60 (2)
H40E	0.196377	0.906008	0.769702	0.189*	0.60 (2)
H40F	0.288578	0.918836	0.728014	0.189*	0.60 (2)
C17B	0.450 (3)	0.2140 (11)	0.364 (2)	0.078 (7)	0.492 (10)
H17B	0.469024	0.225407	0.310347	0.093*	0.492 (10)
C20B	0.361 (10)	−0.003 (3)	0.534 (13)	0.17 (3)	0.37 (19)
H20A	0.324632	−0.003836	0.584783	0.258*	0.37 (19)
H20B	0.421930	−0.008150	0.550824	0.258*	0.37 (19)
H20C	0.343336	−0.034910	0.496940	0.258*	0.37 (19)
C53	0.2151 (14)	0.4419 (13)	0.242 (2)	0.170 (10)	0.5
H53	0.267406	0.454385	0.268713	0.203*	0.5
C54	0.173 (2)	0.4765 (10)	0.1843 (17)	0.169 (10)	0.5
H54	0.203863	0.506632	0.157169	0.203*	0.5
C55	0.087 (2)	0.4679 (12)	0.1655 (16)	0.187 (10)	0.5
H55	0.055147	0.495542	0.133126	0.224*	0.5
C56	0.0454 (15)	0.4182 (16)	0.195 (2)	0.216 (12)	0.5
H56	−0.015053	0.412627	0.184968	0.260*	0.5
C51	0.094 (2)	0.3768 (12)	0.238 (3)	0.214 (12)	0.5
C52	0.179 (2)	0.3887 (13)	0.261 (2)	0.206 (11)	0.5
H52	0.213200	0.360330	0.289331	0.247*	0.5
C20A	0.357 (6)	−0.0027 (18)	0.570 (9)	0.17 (3)	0.63 (19)
H20D	0.398901	−0.030934	0.549149	0.258*	0.63 (19)
H20E	0.300966	−0.008289	0.540742	0.258*	0.63 (19)
H20F	0.349027	−0.008060	0.630419	0.258*	0.63 (19)
O39A	0.3265 (15)	0.8105 (13)	0.7069 (18)	0.102 (8)	0.60 (2)
C39A	0.2499 (18)	0.8360 (11)	0.7152 (17)	0.086 (7)	0.60 (2)
O40A	0.1817 (15)	0.8166 (9)	0.6915 (14)	0.095 (4)	0.60 (2)
C50	0.051 (3)	0.3265 (17)	0.266 (3)	0.238 (18)	0.5
H50A	0.004228	0.337362	0.303933	0.358*	0.5
H50B	0.092790	0.301667	0.295870	0.358*	0.5
H50C	0.026877	0.305478	0.217622	0.358*	0.5

### Bis[(1E,6E)-1,7-bis(4-acetyloxy-3-methoxyphenyl)hepta-1,6-diene-3,5-dionato(1-)-κ2O,O'](methanol)dioxidouranium(VI) toluene monosolvate Atomic displacement parameters (Å^2^)

**Table:**

	*U*^11^	*U*^22^	*U*^33^	*U*^12^	*U*^13^	*U*^23^
U1	0.0993 (4)	0.0338 (2)	0.0371 (2)	0.000	−0.00774 (18)	0.000
O2	0.097 (4)	0.055 (3)	0.049 (3)	0.020 (3)	−0.009 (3)	−0.009 (2)
O1	0.082 (4)	0.039 (2)	0.059 (3)	−0.004 (2)	0.006 (3)	0.000 (2)
O3	0.113 (5)	0.045 (3)	0.052 (3)	0.007 (3)	0.006 (3)	−0.001 (2)
O38	0.117 (6)	0.062 (3)	0.123 (5)	0.021 (4)	−0.042 (5)	−0.034 (3)
C1	0.055 (5)	0.047 (3)	0.056 (4)	−0.006 (3)	−0.006 (3)	0.008 (3)
C3	0.054 (5)	0.055 (3)	0.046 (3)	0.007 (3)	−0.005 (3)	0.005 (3)
C10	0.070 (6)	0.057 (3)	0.067 (5)	−0.011 (4)	−0.001 (4)	0.008 (3)
C31	0.061 (5)	0.061 (3)	0.048 (4)	0.015 (3)	−0.002 (4)	−0.003 (3)
C2	0.080 (6)	0.054 (3)	0.058 (4)	0.001 (4)	0.002 (4)	0.006 (3)
C32	0.060 (5)	0.069 (4)	0.055 (4)	0.018 (4)	−0.018 (4)	−0.015 (3)
C30	0.063 (5)	0.059 (3)	0.050 (4)	0.007 (3)	−0.003 (4)	0.000 (3)
C33	0.070 (6)	0.063 (4)	0.069 (5)	0.017 (4)	−0.020 (4)	−0.019 (3)
C11	0.080 (6)	0.053 (3)	0.067 (5)	−0.009 (4)	−0.023 (4)	0.000 (3)
C37	0.082 (6)	0.087 (5)	0.059 (4)	0.033 (5)	−0.014 (4)	−0.016 (4)
C35	0.084 (6)	0.096 (5)	0.079 (5)	0.040 (5)	−0.036 (5)	−0.041 (4)
C36	0.103 (8)	0.113 (6)	0.059 (5)	0.043 (6)	−0.023 (5)	−0.031 (4)
C34	0.079 (6)	0.068 (4)	0.088 (5)	0.021 (4)	−0.032 (5)	−0.031 (4)
C38	0.106 (9)	0.063 (6)	0.147 (8)	0.001 (5)	−0.026 (8)	−0.006 (6)
C13B	0.069 (14)	0.054 (7)	0.080 (9)	0.006 (8)	−0.001 (10)	0.012 (7)
C14A	0.068 (17)	0.068 (7)	0.110 (16)	−0.024 (8)	−0.015 (10)	0.018 (8)
C15A	0.061 (13)	0.062 (7)	0.118 (13)	−0.025 (8)	−0.012 (9)	0.013 (7)
C14B	0.053 (11)	0.057 (7)	0.092 (9)	0.014 (8)	0.020 (9)	0.020 (6)
C17A	0.11 (2)	0.069 (9)	0.103 (15)	0.002 (11)	−0.014 (13)	0.016 (9)
C13A	0.085 (18)	0.057 (7)	0.085 (16)	−0.017 (8)	−0.035 (11)	0.017 (8)
C12A	0.065 (18)	0.058 (7)	0.103 (16)	−0.019 (11)	−0.044 (10)	0.010 (10)
O19A	0.118 (13)	0.067 (7)	0.153 (15)	−0.028 (7)	−0.005 (11)	0.038 (8)
C15B	0.056 (14)	0.057 (6)	0.100 (11)	−0.009 (7)	−0.002 (10)	0.002 (6)
C16B	0.057 (14)	0.051 (7)	0.098 (11)	−0.004 (8)	0.005 (9)	−0.011 (7)
O19B	0.118 (12)	0.052 (6)	0.146 (15)	−0.004 (6)	0.002 (11)	0.017 (7)
O18A	0.150 (14)	0.070 (7)	0.097 (10)	−0.021 (7)	0.009 (8)	0.012 (6)
C18A	0.13 (2)	0.091 (14)	0.109 (15)	−0.017 (13)	0.016 (12)	0.019 (11)
O18B	0.156 (15)	0.061 (7)	0.101 (9)	0.004 (8)	0.050 (10)	0.022 (6)
C12B	0.08 (2)	0.053 (6)	0.066 (10)	0.018 (9)	−0.015 (11)	0.001 (7)
C18B	0.13 (2)	0.089 (12)	0.092 (13)	0.014 (13)	0.050 (14)	0.024 (9)
C16A	0.13 (2)	0.067 (8)	0.129 (18)	−0.032 (11)	0.009 (14)	−0.003 (9)
O20B	0.128 (14)	0.147 (16)	0.156 (19)	−0.053 (12)	−0.006 (13)	0.021 (13)
O20A	0.145 (14)	0.092 (10)	0.142 (15)	−0.005 (9)	−0.031 (12)	0.019 (10)
C19B	0.135 (16)	0.086 (12)	0.12 (2)	−0.033 (11)	0.015 (15)	0.007 (13)
C19A	0.149 (16)	0.069 (10)	0.13 (2)	−0.013 (9)	−0.027 (16)	0.013 (12)
O39B	0.101 (13)	0.089 (13)	0.075 (11)	0.045 (12)	−0.023 (11)	−0.028 (10)
O40B	0.101 (8)	0.097 (13)	0.086 (6)	0.022 (10)	−0.016 (5)	−0.023 (10)
C39B	0.089 (11)	0.096 (13)	0.073 (11)	0.033 (8)	−0.020 (8)	−0.013 (11)
C40B	0.078 (17)	0.122 (17)	0.18 (2)	0.026 (10)	−0.012 (14)	−0.072 (17)
O4	0.120 (16)	0.048 (5)	0.074 (7)	−0.010 (5)	0.054 (12)	−0.004 (6)
C4	0.18 (3)	0.18 (3)	0.18 (2)	−0.10 (2)	0.11 (2)	−0.11 (2)
C40A	0.078 (17)	0.122 (17)	0.18 (2)	0.026 (10)	−0.012 (14)	−0.072 (17)
C17B	0.097 (19)	0.056 (8)	0.081 (15)	−0.003 (10)	0.002 (14)	−0.008 (8)
C20B	0.199 (19)	0.063 (7)	0.26 (7)	−0.015 (9)	0.03 (4)	0.021 (19)
C53	0.154 (18)	0.129 (17)	0.23 (3)	0.002 (13)	0.061 (17)	−0.079 (16)
C54	0.21 (2)	0.120 (16)	0.18 (2)	−0.029 (15)	0.033 (18)	−0.094 (14)
C55	0.19 (2)	0.17 (2)	0.20 (3)	−0.020 (17)	0.04 (2)	−0.035 (19)
C56	0.21 (2)	0.19 (2)	0.24 (3)	−0.054 (17)	−0.02 (2)	−0.01 (2)
C51	0.22 (2)	0.16 (2)	0.26 (3)	−0.051 (16)	−0.01 (2)	−0.019 (19)
C52	0.20 (2)	0.165 (19)	0.25 (3)	−0.029 (16)	0.01 (2)	−0.034 (19)
C20A	0.199 (19)	0.063 (7)	0.26 (7)	−0.015 (9)	0.03 (4)	0.021 (19)
O39A	0.085 (10)	0.117 (11)	0.104 (15)	0.027 (9)	−0.017 (9)	−0.069 (12)
C39A	0.089 (11)	0.096 (13)	0.073 (11)	0.033 (8)	−0.020 (8)	−0.013 (11)
O40A	0.101 (8)	0.097 (13)	0.086 (6)	0.022 (10)	−0.016 (5)	−0.023 (10)
C50	0.24 (3)	0.15 (3)	0.32 (5)	−0.05 (2)	0.01 (4)	−0.02 (3)

### Bis[(1E,6E)-1,7-bis(4-acetyloxy-3-methoxyphenyl)hepta-1,6-diene-3,5-dionato(1-)-κ2O,O'](methanol)dioxidouranium(VI) toluene monosolvate Geometric parameters (Å, º)

**Table:**

U1—O2	1.772 (6)	C15B—O19B	1.40 (2)
U1—O2^i^	1.772 (6)	C16B—H16B	0.9500
U1—O1^i^	2.345 (5)	C16B—C17B	1.45 (3)
U1—O1	2.345 (5)	O19B—C19B	1.28 (3)
U1—O3^i^	2.351 (5)	O18A—C18A	1.37 (2)
U1—O3	2.351 (5)	C18A—H18A	0.9800
U1—O4^i^	2.567 (9)	C18A—H18B	0.9800
U1—O4	2.567 (9)	C18A—H18C	0.9800
O1—C1	1.279 (8)	O18B—C18B	1.38 (2)
O3—C3	1.283 (9)	C12B—C17B	1.39 (3)
O38—C34	1.372 (12)	C18B—H18D	0.9800
O38—C38	1.434 (14)	C18B—H18E	0.9800
C1—C10	1.468 (10)	C18B—H18F	0.9800
C1—C2	1.379 (11)	C16A—H16A	0.9500
C3—C2	1.370 (11)	O20B—C19B	1.26 (4)
C3—C30	1.468 (10)	O20A—C19A	1.24 (3)
C10—H10	0.9500	C19B—C20B	1.47 (5)
C10—C11	1.286 (11)	C19A—C20A	1.47 (5)
C31—H31	0.9500	O39B—C39B	1.23 (4)
C31—C32	1.462 (10)	O40B—C39B	1.26 (4)
C31—C30	1.295 (10)	C39B—C40B	1.48 (2)
C2—H2	0.9500	C40B—H40A	0.9800
C32—C33	1.380 (11)	C40B—H40B	0.9800
C32—C37	1.384 (11)	C40B—H40C	0.9800
C30—H30	0.9500	O4—H4	0.8400
C33—H33	0.9500	O4—C4	1.47 (3)
C33—C34	1.403 (11)	C4—H4A	0.9800
C11—H11	0.9500	C4—H4B	0.9800
C11—H11A	0.9500	C4—H4C	0.9800
C11—C12A	1.418 (18)	C40A—H40D	0.9800
C11—C12B	1.53 (3)	C40A—H40E	0.9800
C37—H37	0.9500	C40A—H40F	0.9800
C37—C36	1.398 (11)	C40A—C39A	1.466 (19)
C35—C36	1.364 (15)	C17B—H17B	0.9500
C35—C34	1.369 (14)	C20B—H20A	0.9800
C35—O39B	1.42 (4)	C20B—H20B	0.9800
C35—O39A	1.40 (3)	C20B—H20C	0.9800
C36—H36	0.9500	C53—H53	0.9500
C38—H38A	0.9800	C53—C54	1.378 (10)
C38—H38B	0.9800	C53—C52	1.383 (10)
C38—H38C	0.9800	C54—H54	0.9500
C13B—H13B	0.9500	C54—C55	1.375 (10)
C13B—C14B	1.38 (3)	C55—H55	0.9500
C13B—C12B	1.28 (4)	C55—C56	1.395 (10)
C14A—C15A	1.38 (3)	C56—H56	0.9500
C14A—C13A	1.39 (3)	C56—C51	1.392 (10)
C14A—O18A	1.36 (3)	C51—C52	1.391 (10)
C15A—O19A	1.423 (15)	C51—C50	1.41 (4)
C15A—C16A	1.31 (3)	C52—H52	0.9500
C14B—C15B	1.36 (3)	C20A—H20D	0.9800
C14B—O18B	1.37 (2)	C20A—H20E	0.9800
C17A—H17A	0.9500	C20A—H20F	0.9800
C17A—C12A	1.41 (4)	O39A—C39A	1.33 (3)
C17A—C16A	1.48 (3)	C39A—O40A	1.20 (3)
C13A—H13A	0.9500	C50—H50A	0.9800
C13A—C12A	1.41 (4)	C50—H50B	0.9800
O19A—C19A	1.30 (3)	C50—H50C	0.9800
C15B—C16B	1.34 (3)		
			
O2—U1—O2^i^	179.5 (3)	C16B—C15B—C14B	121 (2)
O2^i^—U1—O1^i^	90.9 (2)	C16B—C15B—O19B	120 (2)
O2^i^—U1—O1	89.5 (2)	C15B—C16B—H16B	119.5
O2—U1—O1^i^	89.5 (2)	C15B—C16B—C17B	121 (2)
O2—U1—O1	90.9 (2)	C17B—C16B—H16B	119.5
O2—U1—O3^i^	89.5 (2)	C19B—O19B—C15B	120 (2)
O2^i^—U1—O3	89.5 (2)	C14A—O18A—C18A	117.0 (19)
O2^i^—U1—O3^i^	90.3 (2)	O18A—C18A—H18A	109.5
O2—U1—O3	90.3 (2)	O18A—C18A—H18B	109.5
O2^i^—U1—O4	81.6 (4)	O18A—C18A—H18C	109.5
O2^i^—U1—O4^i^	97.9 (4)	H18A—C18A—H18B	109.5
O2—U1—O4^i^	81.6 (4)	H18A—C18A—H18C	109.5
O2—U1—O4	97.9 (4)	H18B—C18A—H18C	109.5
O1^i^—U1—O1	80.0 (2)	C14B—O18B—C18B	117.4 (15)
O1—U1—O3^i^	152.09 (18)	C13B—C12B—C11	119 (2)
O1—U1—O3	72.05 (17)	C13B—C12B—C17B	128 (2)
O1^i^—U1—O3^i^	72.05 (17)	C17B—C12B—C11	113 (2)
O1^i^—U1—O3	152.09 (18)	O18B—C18B—H18D	109.5
O1^i^—U1—O4^i^	144.9 (4)	O18B—C18B—H18E	109.5
O1—U1—O4	144.9 (4)	O18B—C18B—H18F	109.5
O1^i^—U1—O4	133.6 (3)	H18D—C18B—H18E	109.5
O1—U1—O4^i^	133.6 (3)	H18D—C18B—H18F	109.5
O3^i^—U1—O3	135.9 (2)	H18E—C18B—H18F	109.5
O3^i^—U1—O4^i^	74.0 (3)	C15A—C16A—C17A	117 (3)
O3^i^—U1—O4	62.4 (3)	C15A—C16A—H16A	121.4
O3—U1—O4	74.0 (3)	C17A—C16A—H16A	121.4
O3—U1—O4^i^	62.4 (3)	O19B—C19B—C20B	112 (7)
O4—U1—O4^i^	20.2 (6)	O20B—C19B—O19B	121 (3)
C1—O1—U1	135.0 (4)	O20B—C19B—C20B	127 (6)
C3—O3—U1	134.2 (5)	O19A—C19A—C20A	116 (4)
C34—O38—C38	118.3 (8)	O20A—C19A—O19A	121 (2)
O1—C1—C10	117.5 (7)	O20A—C19A—C20A	124 (4)
O1—C1—C2	124.3 (6)	C39B—O39B—C35	123 (3)
C2—C1—C10	118.2 (7)	O39B—C39B—O40B	118 (3)
O3—C3—C2	123.1 (7)	O39B—C39B—C40B	118 (4)
O3—C3—C30	117.1 (6)	O40B—C39B—C40B	124 (3)
C2—C3—C30	119.8 (7)	C39B—C40B—H40A	109.5
C1—C10—H10	116.5	C39B—C40B—H40B	109.5
C11—C10—C1	126.9 (8)	C39B—C40B—H40C	109.5
C11—C10—H10	116.5	H40A—C40B—H40B	109.5
C32—C31—H31	115.7	H40A—C40B—H40C	109.5
C30—C31—H31	115.7	H40B—C40B—H40C	109.5
C30—C31—C32	128.5 (8)	U1—O4—H4	99.4
C1—C2—H2	116.8	C4—O4—U1	126.4 (17)
C3—C2—C1	126.3 (7)	C4—O4—H4	109.5
C3—C2—H2	116.8	O4—C4—O4^i^	30.1 (9)
C33—C32—C31	118.0 (7)	O4^i^—C4—H4A	108.8
C33—C32—C37	119.5 (7)	O4—C4—H4A	109.5
C37—C32—C31	122.6 (7)	O4^i^—C4—H4B	132.8
C3—C30—H30	117.5	O4—C4—H4B	109.5
C31—C30—C3	125.0 (7)	O4—C4—H4C	109.5
C31—C30—H30	117.5	O4^i^—C4—H4C	82.1
C32—C33—H33	119.9	H4A—C4—H4B	109.5
C32—C33—C34	120.2 (9)	H4A—C4—H4C	109.5
C34—C33—H33	119.9	H4B—C4—H4C	109.5
C10—C11—H11	119.2	H40D—C40A—H40E	109.5
C10—C11—H11A	113.7	H40D—C40A—H40F	109.5
C10—C11—C12A	121.6 (17)	H40E—C40A—H40F	109.5
C10—C11—C12B	132.6 (15)	C39A—C40A—H40D	109.5
C12A—C11—H11	119.2	C39A—C40A—H40E	109.5
C12B—C11—H11A	113.7	C39A—C40A—H40F	109.5
C32—C37—H37	120.1	C16B—C17B—H17B	124.0
C32—C37—C36	119.9 (9)	C12B—C17B—C16B	112 (2)
C36—C37—H37	120.1	C12B—C17B—H17B	124.0
C36—C35—C34	120.8 (8)	C19B—C20B—H20A	109.5
C36—C35—O39B	111 (2)	C19B—C20B—H20B	109.5
C36—C35—O39A	125.9 (16)	C19B—C20B—H20C	109.5
C34—C35—O39B	127.8 (19)	H20A—C20B—H20B	109.5
C34—C35—O39A	112.4 (17)	H20A—C20B—H20C	109.5
C37—C36—H36	119.9	H20B—C20B—H20C	109.5
C35—C36—C37	120.1 (10)	C54—C53—H53	120.8
C35—C36—H36	119.9	C54—C53—C52	118.4 (10)
O38—C34—C33	123.3 (10)	C52—C53—H53	120.8
C35—C34—O38	117.2 (8)	C53—C54—H54	119.7
C35—C34—C33	119.5 (9)	C55—C54—C53	120.6 (10)
O38—C38—H38A	109.5	C55—C54—H54	119.7
O38—C38—H38B	109.5	C54—C55—H55	120.4
O38—C38—H38C	109.5	C54—C55—C56	119.3 (10)
H38A—C38—H38B	109.5	C56—C55—H55	120.4
H38A—C38—H38C	109.5	C55—C56—H56	120.4
H38B—C38—H38C	109.5	C51—C56—C55	119.2 (10)
C14B—C13B—H13B	120.7	C51—C56—H56	120.4
C12B—C13B—H13B	120.7	C56—C51—C50	118 (3)
C12B—C13B—C14B	119 (2)	C52—C51—C56	119.7 (10)
C15A—C14A—C13A	120 (3)	C52—C51—C50	121 (3)
O18A—C14A—C15A	116 (2)	C53—C52—C51	119.8 (10)
O18A—C14A—C13A	123 (3)	C53—C52—H52	120.1
C14A—C15A—O19A	119 (2)	C51—C52—H52	120.1
C16A—C15A—C14A	122 (2)	C19A—C20A—H20D	109.5
C16A—C15A—O19A	119 (2)	C19A—C20A—H20E	109.5
C15B—C14B—C13B	120 (2)	C19A—C20A—H20F	109.5
C15B—C14B—O18B	116.9 (18)	H20D—C20A—H20E	109.5
O18B—C14B—C13B	123.5 (19)	H20D—C20A—H20F	109.5
C12A—C17A—H17A	117.4	H20E—C20A—H20F	109.5
C12A—C17A—C16A	125 (2)	C39A—O39A—C35	117.5 (19)
C16A—C17A—H17A	117.4	O39A—C39A—C40A	113 (2)
C14A—C13A—H13A	117.5	O40A—C39A—C40A	122 (2)
C14A—C13A—C12A	125 (3)	O40A—C39A—O39A	125 (2)
C12A—C13A—H13A	117.5	C51—C50—H50A	109.5
C17A—C12A—C11	127 (3)	C51—C50—H50B	109.5
C13A—C12A—C11	122 (3)	C51—C50—H50C	109.5
C13A—C12A—C17A	110.6 (18)	H50A—C50—H50B	109.5
C19A—O19A—C15A	121 (2)	H50A—C50—H50C	109.5
C14B—C15B—O19B	118 (2)	H50B—C50—H50C	109.5
			
U1—O1—C1—C10	−171.1 (5)	C14A—C15A—O19A—C19A	74 (3)
U1—O1—C1—C2	9.9 (12)	C14A—C15A—C16A—C17A	0 (4)
U1—O3—C3—C2	−24.7 (12)	C14A—C13A—C12A—C11	−176 (3)
U1—O3—C3—C30	157.3 (5)	C14A—C13A—C12A—C17A	1 (5)
U1—O4—C4—O4^i^	−81.2 (15)	C15A—C14A—C13A—C12A	−6 (5)
O1—C1—C10—C11	9.0 (12)	C15A—C14A—O18A—C18A	176 (2)
O1—C1—C2—C3	3.7 (13)	C15A—O19A—C19A—O20A	10 (4)
O3—C3—C2—C1	3.5 (13)	C15A—O19A—C19A—C20A	−174 (6)
O3—C3—C30—C31	−0.1 (12)	C14B—C13B—C12B—C11	179 (3)
C1—C10—C11—C12A	−177 (2)	C14B—C13B—C12B—C17B	−3 (7)
C1—C10—C11—C12B	173 (3)	C14B—C15B—C16B—C17B	−5 (4)
C10—C1—C2—C3	−175.3 (7)	C14B—C15B—O19B—C19B	90 (3)
C10—C11—C12A—C17A	9 (6)	C13A—C14A—C15A—O19A	178 (3)
C10—C11—C12A—C13A	−176 (3)	C13A—C14A—C15A—C16A	5 (4)
C10—C11—C12B—C13B	−4 (6)	C13A—C14A—O18A—C18A	−13 (4)
C10—C11—C12B—C17B	178 (2)	C12A—C17A—C16A—C15A	−6 (5)
C31—C32—C33—C34	179.2 (8)	O19A—C15A—C16A—C17A	−173 (2)
C31—C32—C37—C36	−178.6 (8)	C15B—C14B—O18B—C18B	−179 (2)
C2—C1—C10—C11	−171.9 (8)	C15B—C16B—C17B—C12B	3 (5)
C2—C3—C30—C31	−178.1 (8)	C15B—O19B—C19B—O20B	3 (5)
C32—C31—C30—C3	−177.6 (7)	C15B—O19B—C19B—C20B	−172 (9)
C32—C33—C34—O38	−179.7 (8)	C16B—C15B—O19B—C19B	−98 (3)
C32—C33—C34—C35	−0.1 (14)	O19B—C15B—C16B—C17B	−177 (3)
C32—C37—C36—C35	−0.9 (15)	O18A—C14A—C15A—O19A	−10 (3)
C30—C3—C2—C1	−178.6 (8)	O18A—C14A—C15A—C16A	177 (2)
C30—C31—C32—C33	−174.3 (8)	O18A—C14A—C13A—C12A	−177 (3)
C30—C31—C32—C37	6.0 (14)	O18B—C14B—C15B—C16B	−176 (2)
C33—C32—C37—C36	1.7 (13)	O18B—C14B—C15B—O19B	−5 (3)
C11—C12B—C17B—C16B	179 (3)	C12B—C13B—C14B—C15B	1 (4)
C37—C32—C33—C34	−1.2 (13)	C12B—C13B—C14B—O18B	−180 (3)
C35—O39B—C39B—O40B	12 (7)	C16A—C15A—O19A—C19A	−113 (3)
C35—O39B—C39B—C40B	−166 (4)	C16A—C17A—C12A—C11	−179 (3)
C35—O39A—C39A—C40A	−175 (2)	C16A—C17A—C12A—C13A	5 (5)
C35—O39A—C39A—O40A	8 (5)	O39B—C35—C36—C37	174.6 (19)
C36—C35—C34—O38	−179.5 (9)	O39B—C35—C34—O38	6 (3)
C36—C35—C34—C33	0.9 (15)	O39B—C35—C34—C33	−173 (2)
C36—C35—O39B—C39B	−98 (4)	C53—C54—C55—C56	−11 (2)
C36—C35—O39A—C39A	−103 (3)	C54—C53—C52—C51	−15 (5)
C34—C35—C36—C37	−0.4 (16)	C54—C55—C56—C51	−4 (2)
C34—C35—O39B—C39B	76 (5)	C55—C56—C51—C52	8 (5)
C34—C35—O39A—C39A	88 (3)	C55—C56—C51—C50	−180 (4)
C38—O38—C34—C33	−6.9 (14)	C56—C51—C52—C53	1 (5)
C38—O38—C34—C35	173.5 (9)	C52—C53—C54—C55	20 (4)
C13B—C14B—C15B—C16B	3 (3)	O39A—C35—C36—C37	−168.9 (14)
C13B—C14B—C15B—O19B	175 (2)	O39A—C35—C34—O38	−9.5 (16)
C13B—C14B—O18B—C18B	1 (3)	O39A—C35—C34—C33	170.8 (12)
C13B—C12B—C17B—C16B	1 (7)	C50—C51—C52—C53	−171 (4)

Symmetry code: (i) −*x*+1, *y*, −*z*+1/2.

### Bis[(1E,6E)-1,7-bis(4-acetyloxy-3-methoxyphenyl)hepta-1,6-diene-3,5-dionato(1-)-κ2O,O'](methanol)dioxidouranium(VI) toluene monosolvate Hydrogen-bond geometry (Å, º)

**Table:**

*D*—H···*A*	*D*—H	H···*A*	*D*···*A*	*D*—H···*A*
O4—H4···O40*A*^ii^	0.84	2.33	2.99 (2)	136
O4—H4···O40*B*^ii^	0.84	2.67	3.23 (4)	125
C40*A*—H40*D*···O20*B*^iii^	0.98	2.46	3.25 (4)	137
C30—H30···O2^iii^	0.95	2.58	3.436 (9)	150

Symmetry codes: (ii) *x*+1/2, −*y*+3/2, *z*−1/2; (iii) *x*, −*y*+1, *z*+1/2.
